# Biotechnological production of glycolic acid and ethylene glycol: current state and perspectives

**DOI:** 10.1007/s00253-019-09640-2

**Published:** 2019-02-01

**Authors:** Laura Salusjärvi, Sami Havukainen, Outi Koivistoinen, Mervi Toivari

**Affiliations:** Solutions for Natural Resources and Environment, VTT Technical Research Centre of Finland Ltd, Tietotie 2, P.O. Box 1000, 02044 VTT Espoo, Finland

**Keywords:** Glycolic acid, Ethylene glycol, D-xylose, Glyoxylate shunt, D-xylulose-1-phosphate pathway, D-ribulose-1-phosphate pathway, L-xylulose-1-phosphate pathway, Dahms pathway, Serine pathway, Metabolic engineering, Biotechnology, Biorefinery

## Abstract

Glycolic acid (GA) and ethylene glycol (EG) are versatile two-carbon organic chemicals used in multiple daily applications. GA and EG are currently produced by chemical synthesis, but their biotechnological production from renewable resources has received a substantial interest. Several different metabolic pathways by using genetically modified microorganisms, such as *Escherichia coli*, *Corynebacterium glutamicum* and yeast have been established for their production. As a result, the yield of GA and EG produced from sugars has been significantly improved. Here, we describe the recent advancement in metabolic engineering efforts focusing on metabolic pathways and engineering strategies used for GA and EG production.

## Introduction

**Fig. 1:**
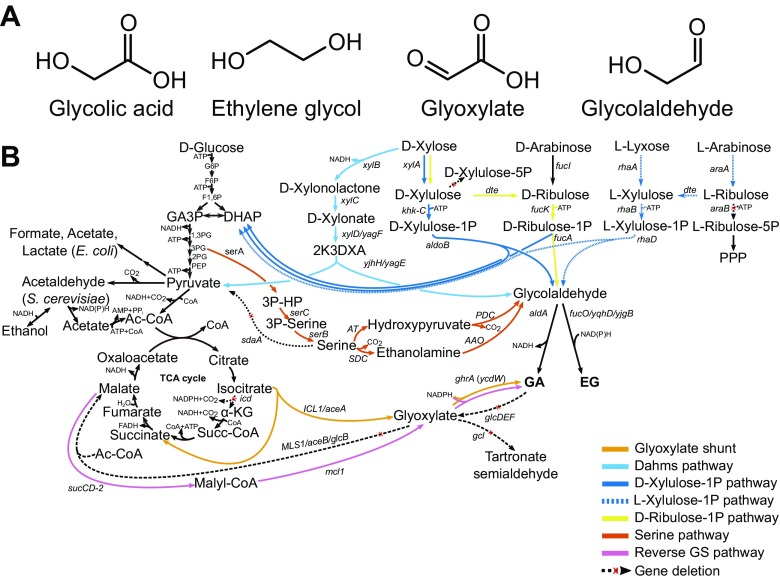
Overview of glycolic acid and ethylene glycol production pathways. **a** The structures of the products and their immediate precursors. **b** Pathways that have been used for the production of glycolic acid or ethylene glycol in metabolically engineered microorganisms. G6P glucose-6-phosphate, F6P fructose-6-phosphate, F1,6P fructose-1,6-bisphosphate, GA3P glyceraldehyde 3-phosphate, DHAP dihydroxyacetone phosphate, 1,3PG 1,3-bisphosphoglycerate, 3PG 3-phosphoglycerate, PEP phosphoenolpyruvate, 3P-HP 3-phospho-hydroxypyruvate, PPP pentose phosphate pathway, *glcDEF* glycolate oxidase, *gcl* glycolate carboligase. For descriptions of the enzymes of the individual pathways, refer to Figs. 2, 3, 4 and 5

Glycolic acid (GA) is a small two-carbon α-hydroxy acid (Fig. 1a) with both alcohol and acid groups (pKa 3.83). Textile industry uses GA as a dyeing and tanning agent, in food industry, it is used as a flavour and preservative and in the pharmaceutical industry as a skin care agent. It is also used in industrial and household cleaning agents and adhesives, and it is often included into emulsion polymers, solvents and additives for ink and paint in order to improve flow properties and gloss (Yunhai et al. 2006). GA can also be converted to biodegradable polymer (PGA) with good mechanical properties (Gädda et al. 2014), and it is used together with lactic acid to produce a co-polymer (PLGA) for different medical applications. The GA market size was valued at US$159.6 million in 2015, and it has been constantly growing driven by increasing use of GA in cosmetic products and household cleaning agents. The market is expected to reach US$415.0 million by 2024 (https://www.grandviewresearch.com/press-release/global-glycolic-acid-market).

GA is naturally produced by some chemolithotrophic iron- and sulphur-oxidizing bacteria (Nancucheo and Johnson 2010) or from glycolonitrile by hydrolyzation by nitrilase enzyme activity of *Alcaligenes* sp. ECU0401 (He et al. 2010). GA is also produced by a variety of yeast and acetic acid bacteria from EG by oxidation (Kataoka et al. 2001; Wei et al. 2009). As an example, optimisation of the *Gluconobacter oxydans* cell catalysis and development of the entire bioprocess has enabled production of 575.4 g GA from 497.2 g EG by subsequent alcohol and aldehyde oxidation reactions at the recovery rate of 98.9% (Hua et al. 2018). However, due to relatively high price of EG, GA currently in the market is produced chemically from petrochemical resources mainly in a process where formaldehyde is carbonylated by synthesis gas or treated with carbon monoxide and water (Drent et al. 2002). Other production routes include electrolytic reduction of oxalic acid and hydrolysis of glycolonitrile (Miltenberger 2000).

Ethylene glycol (EG) is a two-carbon dihydroxy alcohol (Fig. 1a). The most noticeable uses of EG are as an antifreeze agent in multiple applications and as precursor for the polymer poly(ethylene terephthalate)(PET). It is also widely used in chemical industries e.g. in paints and resins (http://www.meglobal.biz/monoethylene-glycol). EG is predominantly produced either by the hydration of ethylene oxide using either thermal or catalytic reaction or from ethylene oxide by carbonation and subsequent hydrolyzation with a base catalyst. Direct EG production from renewable resources such as plant-derived materials through chemical catalysis has been reported, and small amount of EG in the market is produced by dehydration of biobased ethanol (Ji et al. 2009; Pang et al. 2011; Fan et al. 2013). These technologies, however, rely on chemical processes that require relatively high operating costs. EG is produced in large volumes, 28 million tons globally, and its demand is expected to almost double in the next 20 years (http://www.bioplasticsmagazine.com/en/news/meldungen/20180612Avantium-to-build-bio-MEG-demonstration-plant-in-the-Netherlands.php).

**Fig. 2:**
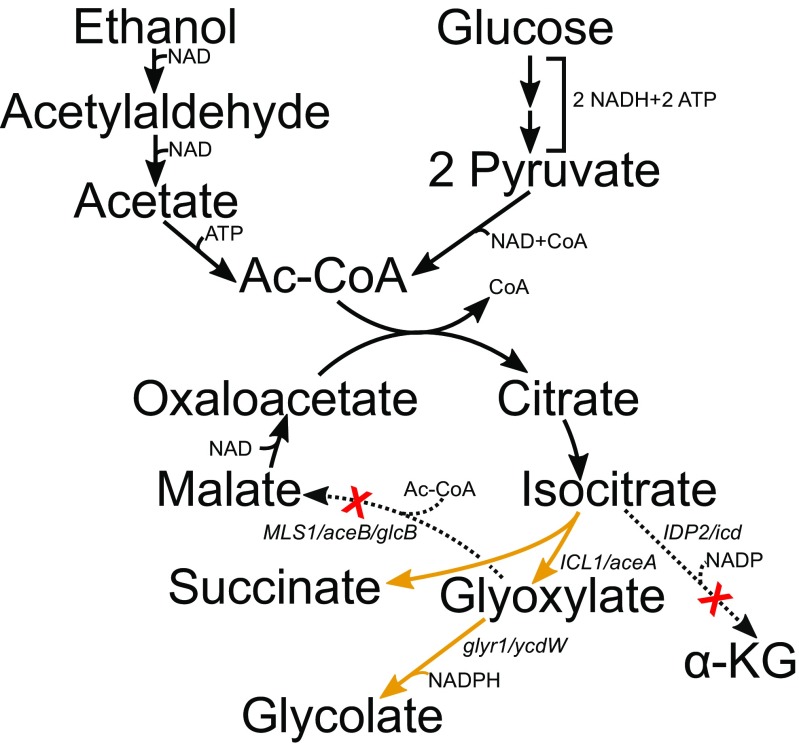
Glyoxylate shunt pathway for GA production from D-glucose or ethanol, with the latter only applicable to yeast hosts. *IDP2/icd* isocitrate dehydrogenase, *ICL1/aceA* isocitrate lyase, *MLS1/aceB/glcB* malate synthase, *glyr1/ycdW* glyoxylate reductase

**Fig. 3:**
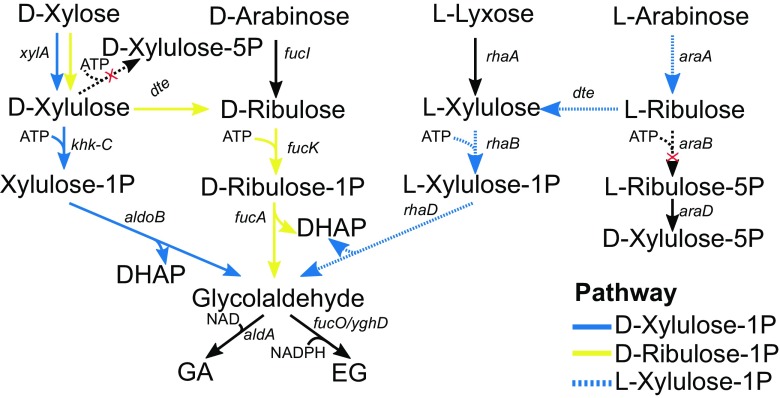
Pentose-1-phosphate pathways for GA or EG production. The three depicted pathways have either D-xylulose-1-phosphate (blue lines), D-ribulose-1-phosphate (yellow) or L-xylulose-1-phosphate (blue, dashed) as the intermediate. *xylA* D-xylose isomerase, *khk-C* ketohexokinase, aldoB fructose-1,6-bisphosphate aldolase, *fucI* L-fucose isomerase, *fucK* L-fuculokinase, *fucA* L-fuculose phosphate aldolase, *rhaA* L-rhamnose isomerase, *rhaB* L-rhamnulokinase, *rhaD* L-rhamnose 1-phosphate aldolase, *araA* L-arabinose isomerase, *araB* ribulokinase, *araD* L-ribulose-5-phosphate aldolase, *aldA* glycolaldehyde dehydrogenase, *fucO*/*yqhD* glycolaldehyde reductase

**Fig. 4:**
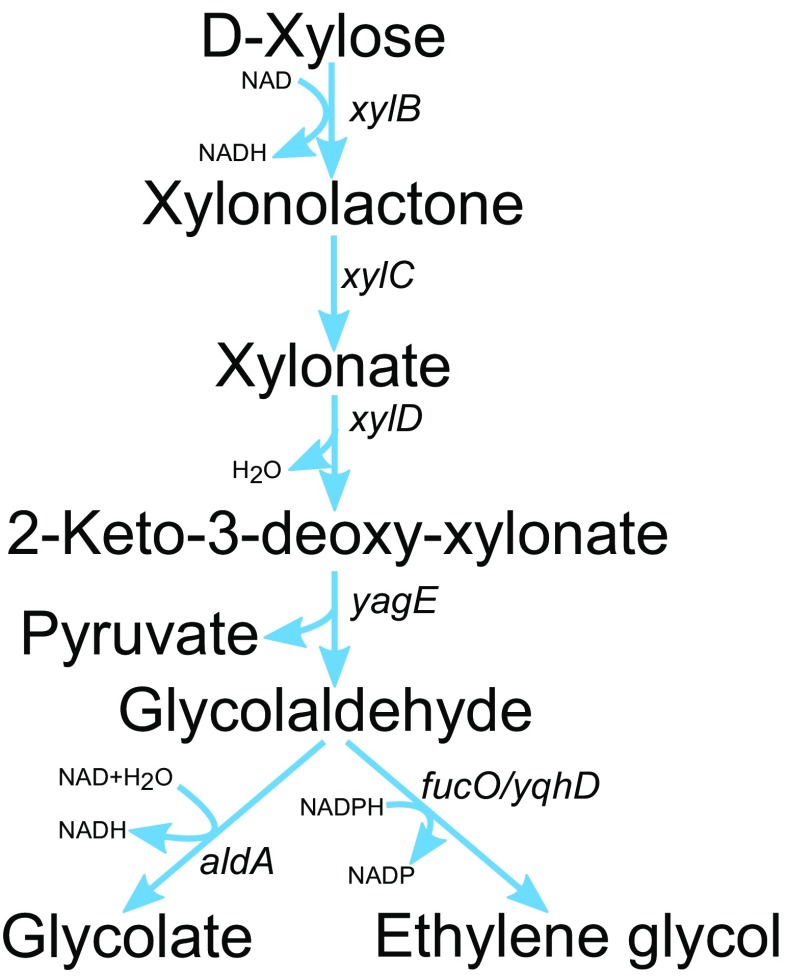
Dahms pathway for GA or EG production from D-xylose. *xylB* D-xylose dehydrogenase, *xylC* D-xylonolactone lactonase, *xylD* D-xylonate dehydratase, *yagE* aldolase, *aldA* glycolaldehyde dehydrogenase, *fucO*/*yqhD* glycolaldehyde reductase

**Fig. 5:**
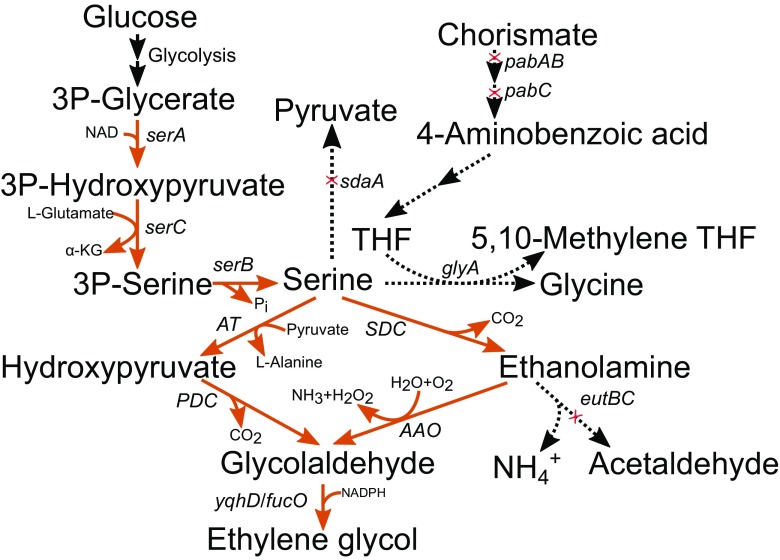
Serine pathway that has been used for ethylene glycol production. There are two options from serine to glycolaldehyde: via aminotransferase and pyruvate decarboxylase (hydroxypyruvate intermediate) or via serine decarboxylase and amino acid oxidase (ethanolamine intermediate). Activity of serine-consuming serine hydroxymethyltransferase *glyA* has been attenuated in the literature by the deletion of 4-aminobenzoic acid biosynthetic genes (*pabABC*) since the *glyA* deletion mutant is inviable. *sera* 3-phosphoglycerate dehydrogenase, *serC* phosphoserine aminotransferase, *serB* phosphoserine phosphatase, *sdaA* L-serine dehydratase, *pabAB* aminodeoxychorismate synthase, *pabC* aminodeoxychorismate lyase, *glyA* serine hydroxymethyltransferase, *AT* aminotransferase, *PDC* pyruvate decarboxylase, *SDC* serine decarboxylase, *AAO* amino acid oxidase, *eutBC* ethanolamine ammonia lyase

There are no known natural microbial pathways to directly produce GA or EG from renewable and relatively cheap feedstocks. Therefore, several synthetic pathways for microbial production of GA or EG from pentose or hexose sugars or ethanol have been established by using either *E. coli*, *C. glutamicum*, *Saccharomyces cerevisiae* or *Kluyveromyces lactis* as hosts (Table 1 and Figs. 1, 2, 3, 4 and 5). This review summarises and discusses the different metabolic engineering approaches and achievements for GA and EG biosynthesis.

**Table 1:** Selected metabolically engineered strains for ethylene glycol and glycolic acid production. GS, glyoxylate shunt; R1P, D-ribulose-1-phosphate pathway; DMS, the Dahms pathway; RGBP, reverse glyoxylate bypass; X1P, L-xylulose-1-phosphate pathway; SER, serine pathway; GA, glycolic acid; EG, ethylene glycol

Genotype	c, g/L	g/g	mol/mol	% theor	g/L/h	C-src	Path	Prod	Ref
*E. coli* Mgly434 MG1655(DE3)Δ*ldhA* Δ*glcB* Δ*aceB* Δ*aldA ycdW aceA aceK gltA*	65.5	0.77	1.83	90	0.85	Glu	GS	GA	(Deng et al. 2018)
*E. coli* BW25113 *ΔaceB ΔglcB*::WAK* (evolved)	56.4	0.52	1.23	62	~ 0.47	Glu	GS	GA	(Deng et al. 2015)
*E. coli* MG1655 *icd*::Cm *ΔaceB Δgcl ΔglcDEFGB ΔaldA ΔiclR Δedd + eda ΔpoxB ΔackA + pta ΔarcA*::Km *ycdW aceA*	52.2	0.38	0.90	45	~ 1.33	Glu	GS	GA	(Dischert and Soucaille 2012)
*E. coli* MG1655 *ΔxylB ΔglcD dte fucA fucK aldA*	44.0	0.44	0.87	87	0.92	Xyl	R1P	GA	(Pereira et al. 2016a)
*E. coli* MG1655 *ΔxylB ΔglcD ΔglcB ΔaceB Δgcl dte fucA fucK aldA aceA aceK ycdW*	41.0	0.62	1.22	61	~ 0.36	Xyl	R1P + GS	GA	(Pereira et al. 2016a)
*K. lactis* H3954 *(pGLYR1, mls1-Δ1, idp2-Δ1)*	15.0	0.52	0.32	32	0.11	EtOH	GS	GA	(Koivistoinen et al. 2013)
*C. glutamicum ΔaceB icd*_*GTG*_ *ycdW*	5.3	0.18	–	–	0.10	Glu + Ace	GS	GA	(Zahoor and Otten 2014)
*E. coli* GA19 ∆*xylAB* ∆*glcD xdh yagE yagF aldA ycdW aceA aceK*	4.57	0.46	0.91	92	–	Xyl	DMS-GS-RGBP	GA	(Cabulong et al. 2018)
*E. coli* MG1655 *ΔxylB ΔglcD khk-c aldoB aldA*	4.3	0.46	0.90	90	–	Xyl	X1P	GA	(Cam et al. 2016)
*E. coli* Pen979 *ΔaceB ΔglcDEFGB Δgcl Δedd-eda ΔiclR ΔarcA Δicd ΔxylB galPproD ghrA aceA khk-c aldoB aldA*	3.73	0.63	–	–	–	Xyl + Glu	X1P + GS	GA	(Alkim et al. 2016)
*S. cerevisiae* H4099 (*MATα*, *ura3–52 HIS3*, *leu2–3/112*, *TRP1*, *MAL2–8* c, SUC2, gre3::*xylB, fra2::HygR,* xylD, yagE, aldA, ldhL)	1.0	–	–	–	–	Xyl + Glu	DMS	GA	(Salusjärvi et al. 2017)
*S. cerevisiae* H3994 (MATα his3 leu2 trp1 ura3::XYL1-XYL2 xks1::XKS1 MAL2–8c SUC2, pGLYR1, mls1-Δ1, dal7-Δ1, idp2-Δ1::ICL1, reg1-Δ1)	0.91	–	–	–	–	Xyl + EtOH	GS	GA	(Koivistoinen et al. 2013)
*E. coli* WL3110 (*pTacxylBC*-P1-anti-*xylB*)	108.2	0.36	0.87		2.25	Xyl	DMS	EG	(Chae et al. 2018)
*E. coli* BL21(DE3)*△arcA△aldA*/ pETDuet1-*yjhH-xdh-xylC*/pACYCDuet1-*fucO-yjhG*	72.0	0.40	0.97	–	1.39	Xyl	DMS	EG	(Wang et al. 2018)
*E. coli* MG1655 *ΔxylB ΔaldA dte fucA fucK fucO*	40	0.35	0.85	85	0.58	Xyl	R1P	EG	(Pereira et al. 2016a)
*E. coli* MG1655 *ΔxylB ΔaldA khk-C aldoB fucO*	20	0.38	0.91	91	0.37	Xyl	X1P	EG	(Alkim et al. 2015)
*E. coli* W3110(DE3) *ΔxylA xdh yqhD*	11.7	0.29	0.70	70	0.24	Xyl	DMS	EG	(Liu et al. 2013)
*E. coli* MG1655 *ΔaraB ΔaldA ΔxylB dte rhaB rhaD fucA fucK fucO*	10.5	0.35	–	–	–	L-Ara + D-Xyl	LX1P + R1P	EG	(Pereira et al. 2016a)
*E. coli* W3110 ΔxylABΔaldAΔyjgBpKMX and pTrcHis2A_yjgB	7.72	0.39	0.95	95	–	Xyl	DMS	EG	(Cabulong et al. 2017)
*E. coli* MG1655(DE3) *ΔaldA serA:317 serB serC fucO sdc*_*V.carteri*_*aao*	4.1	0.14	0.41	20	–	Glu	SER	EG	(Pereira et al. 2016b)
*C. glutamicum Mdlc yqhD Sdc* ASAO yqhD yqhD	3.5	0.09	0.25	13	–	Glu	SER	EG	(Chen et al. 2017)
*S. cerevisiae xks1Δ* Bs*XI* RnKHK *FBA1* CD	0.5	0.01	0.03	3	–	Xyl	X1P	EG	(Chomvong et al. 2016)
*S. cerevisiae* H4099 (*MATα*, *ura3–52 HIS3*, *leu2–3/112*, *TRP1*, *MAL2–8* c, SUC2, gre3::*xylB, fra2::HygR,* xylD)	0.014	–	–	–	–	Xyl + Glu	DMS	EG	(Salusjärvi et al. 2017)
*S. cerevisiae (PeXYLA)*_*47*_ *RPE1 RKI1 TKL1 PsTAL1 PFK1 PFK2*	4.05	–	–	–	–	Xyl + Glu	X1P	EG	(Uranukul et al. 2018)

## Synthetic pathways for glycolic acid and ethylene glycol production

### Modified glyoxylate shunt pathway

The naturally occurring glyoxylate shunt (GS) is the most studied of the metabolic pathways for GA production (Fig. 2). The glyoxylate cycle consists of the upper part of the TCA cycle from malate to isocitrate and the two unique reactions of GS that include the formation of glyoxylate and succinate from isocitrate by isocitrate lyase and the formation of malate from glyoxylate and acetyl-CoA by malate synthase. GA is formed from glyoxylate by one reaction catalysed by glyoxylate reductase (Fig. 2). In terms of carbon sources, GS route is versatile as it involves metabolites of the central carbon metabolism such as pyruvate, oxaloacetate and citrate. So far, D-glucose, D-xylose, ethanol and acetate have been used for GA production via this pathway by using hosts *E. coli*, *C. glutamicum*, *S. cerevisiae* or *K. lactis* (Table 1).

The most common genetic modifications involved in the use of GS aim at accumulation of the TCA cycle metabolite isocitrate and include deletion of genes encoding isocitrate dehydrogenase (or attenuation of its activity) and malate synthase. In order to enhance the flux from isocitrate to GA, isocitrate lyase and glyoxylate reductase encoding genes have been overexpressed (Fig. 2). These modifications enabled GA production in all organisms tested (Koivistoinen et al. 2013; Zahoor and Otten 2014; Deng et al. 2015), but titers higher than couple of grams per liter require still further, rather extensive modification of strains. In *E. coli*, these included adaptive evolution and optimisation of transcription levels of genes encoding e.g. glyoxylate reductase and isocitrate lyase. Further, improvement in GA yield was achieved by deletion of side pathways such as lactate and acetate production and glycolate oxidases and aldehyde dehydrogenase converting GA to glyoxylate or glycolaldehyde, respectively (Fig. 1b) (Deng et al. 2018).

Yeast or *C. glutamicum* could be better suited for the production of GA than *E. coli* because they have higher resistance to low pH and to the product itself. However, work on *S. cerevisiae* and *K. lactis* (Koivistoinen et al. 2013) showed that engineering of yeast to produce GA from sugars via GS was not trivial. Both engineered yeast species showed better GA production when cultivated in the presence of ethanol. Similarly, a *C. glutamicum* strain, which had modifications in the TCA cycle and GS, produced GA only from acetate, whereas D-glucose only supported growth (Zahoor and Otten 2014). This may be related to strong repression of the TCA cycle and GS in the presence of D-glucose in these organisms (Koivistoinen et al. 2013). It is also possible that different compartmentalisation of TCA cycle and GS enzymes into mitochondria and cytosol in yeast makes the pathway less efficient compared with *E. coli*.

D-glucose repression plays a role also in GA production via GS in *E. coli* as D-glucose-induced dephosphorylation regulates the activity of isocitrate dehydrogenase. A common mean to keep isocitrate dehydrogenase phosphorylated and thus inactive even when D-glucose is available is to overexpress isocitrate dehydrogenase kinase/phosphatase aceK. Because of inactivation of isocitrate dehydrogenase, the *E. coli* strains engineered for GA production via GS had low fitness due to weakness of the TCA cycle and imbalance between the TCA cycle and GS reactions. Overexpression of citrate synthase accelerated the TCA cycle and alleviated this problem. However, in bioreactor, low biomass and sensitivity of cells to GA and high concentrations of D-glucose caused problems making it necessary carefully control carbon/nitrogen ratio and D-glucose feeding. However, so far, the highest GA production titers from GS were reported (Table 1) (65.5 g/L, 90% of the theoretical yield) (Deng et al. 2018).

In addition to D-glucose repression of TCA cycle and GS, a general problem in all pathways employing GS for GA production relates to NADPH preference of the glyoxylate reductase enzymes that generates redox imbalance in cells. Attenuation of isocitrate dehydrogenase reaction reinforces the imbalance as it results in further reduction of NADPH availability. Expression of NADH-utilizing glyoxylate reductase would be beneficial for GA production, but up to date, good enzyme candidates have not been reported.

### D-xylulose-1-phosphate pathway

The synthetic D-xylulose-1-phosphate pathway proceeds via isomerisation of D-xylose to D-xylulose that is phosphorylated to D-xylulose-1-phosphate (X1P) that is then aldolytically cleaved to yield glycolaldehyde and dihydroxyacetone phosphate (DHAP) (Fig. 3). In this pathway, phosphorylation of D-xylulose takes place at position 1 in contrast to natural D-xylose assimilation pathway where D-xylulose is phosphorylated to D-xylulose-5-phosphate. Both glycolaldehyde and DHAP can be converted to GA, if the GS is also engineered for conversion of DHAP to GA. Reduction of glycolaldehyde by glycolaldehyde reductase results in EG production. The pathway has been used for both GA and EG production from D-xylose by *E. coli* and for EG production by *S. cerevisiae* (Alkim et al. 2015; Alkim et al. 2016; Chomvong et al. 2016; Cam et al. 2016).

For GA production in *E. coli*, the human or rat D-xylulose-1-kinase and human D-xylulose-1-phosphate aldolase were selected from couple of enzyme candidates tested for D-xylulose phosphorylation and X1P cleavage (Chomvong et al. 2016; Cam et al. 2016). After some additional modifications, including blockage of D-xylulose conversion to D-xylulose-5-phosphate by deletion of endogenous xylulokinase-encoding gene and prevention of GA consumption by deletion of *glcD* encoding the subunit of glycolate oxidase, 4.3 g/L of GA with yield of 0.46 g/g (90% of the theoretical maximum) was obtained (Cam et al. 2016).

Combination of the X1P and glyoxylate bypass would theoretically increase the GA yield from D-xylose by 20%. When tested in practice in *E. coli*, simultaneous expression of these pathways did not increase GA production from D-xylose, possibly due to low growth of the engineered strain. Addition of D-glucose in media, however, increased GA yield 27% in strain with both pathways compared to the presence of the glyoxylate pathway alone. D-xylose utilisation in medium with both D-xylose and D-glucose as carbon sources was further improved by switching the native promoter of sugar permease galP to strong and constitutive proD promoter. However, deletion of isocitrate dehydrogenase and consequent inactivation of the oxidative TCA cycle in this strain resulted in need of amino acid supplementation of growth media (Alkim et al. 2016).

Production EG via X1P pathway in *E. coli* requires deletion of *aldA* to prevent GA formation and overexpression of *fucO*-encoding glycolaldehyde reductase in order to convert glycolaldehyde to EG (Alkim et al. 2015). With these modifications, 20 g/L EG from D-xylose with a yield of 0.38 g/g was produced. Cultivation under aerobic conditions was important for EG production as decreasing oxygen supply resulted in increased acetate and succinate production that decreased both biomass and EG yield. Overall, carbon was also lost as DHAP that was not converted to EG.

The pathway was also expressed in *S. cerevisiae* for EG and ethanol production under anaerobic conditions (Chomvong et al. 2016). The expression of D-xylose isomerase-encoding gene from *Bacteroides stercoris* enabled D-xylose utilisation of the strain. Endogenous xylulokinase-encoding gene *XKS1* was deleted in order to prevent conversion of D-xylulose to D-xylulose-5-phosphate. Ketohexokinase from rat liver phosphorylated D-xylulose to D-xylulose-1-phosphate that was further converted to glycolaldehyde and DHAP by endogenous Fba1 activity. Glycolaldehyde was reduced to EG by endogenous Gre2 and Adh1 activities, and DHAP was further metabolised via glycolysis. However, only small amount of EG was produced (0.5 g/L) due to several reasons. The conversion of the D-xylose to D-xylulose by D-xylose isomerase was inefficient, and also, the activities of the other pathway enzymes were low in yeast. Moreover, the pathway created a redox imbalance by producing excess NAD^+^ and NADP^+^, and there was a potential ATP deficiency due to phosphorylation of D-xylulose by ketohexokinase early on the pathway. In addition, metabolites from the X1P pathway were leaking to pentose phosphate pathway and D-xylulose-1-phosphate was likely consumed by unknown endogenous enzyme activities.

### D-ribulose-1-phosphate and L-xylulose-1-phosphate pathways

D-ribulose-1-phosphate pathway employs the native D-arabinose and L-lyxose catabolic pathways in *E. coli* for production of GA or EG from D-arabinose or D-xylose. The enzymes of the pathway FucI, FucK and FucA not only cleave D-arabinose into glycolaldehyde and DHAP but also L-fucose into lactaldehyde and DHAP. In the synthetic GA or EG pathways, the FucO and AldA enzymes catalyse the reduction and oxidation of glycolaldehyde into EG and GA, respectively (Fig. 3). Similar to X1P pathway, both glycolaldehyde and DHAP can be converted to GA while EG can be produced only by reduction of glycolaldehyde. The pathway can be modified for use of D-xylose as a carbon source by expression of D-tagatose epimerase that interconverts D-xylulose and D-ribulose and also L-ribulose and L-xylulose (Fig. 3) (Stephanopoulos et al. 2013; Pereira et al. 2016a).

Pereira et al. (2016a) introduced this pathway into *E. coli* to produce 40 g/L EG from D-xylose at a yield of 0.35 g/g and 20 g/L EG from L-arabinose at a yield of 0.38 g/g. Further strain engineering resulted in production of 10.5 g/L EG from D-xylose and L-arabinose simultaneously. Deletion of *glcD* to prevent oxidation of GA to glyoxylate and overexpression of *aldA* for oxidation of glycolaldehyde to GA led to production of 44 g/L GA in a batch fermentation with yield of 0.44 g/g D-xylose (Pereira et al. 2016a). In this strain, only the two carbons of D-ribulose-1-phosphate were utilised for GA production and as in case of X1P pathway, additional use of the remaining three-carbons would further improve the yield of GA. Therefore, also the GS pathway was engineered into the strain resulting in production of 41 g/L GA and a yield of 0.62 g/g in a batch bioreactor (Pereira et al. 2016a). Approximately, 65 g/L D-xylose was used as the substrate and all D-xylose was consumed after 85 h. The combination of pathways for both two- and three-carbon compounds generated approximately the same titer, but the yield increased roughly 40% compared to the two-carbon pathway.

A recent article by Uranukul et al. (2018) described the application of R1P pathway for EG production in *S. cerevisiae*. The pathway was expressed in a D-xylose-utilizing strain that had been evolutionally engineered to have increased growth rates on D-xylose, and from which all copies of D-xylulose kinase genes had been deleted (adaptive evolution had resulted in multiple copies of *P. stipitis XYL3* and endogenous *XKS1* genes in the genome). However, the authors noticed that the deletion of the copies of *XYL3* gene was enough to cause EG production from D-xylose during microaerobic, high cell-density cultivation even in the absence of the R1P pathway. The authors therefore investigated the endogenous enzyme activities which were responsible for EG production and found out that the most likely candidates for converting D-xylulose to glycolaldehyde and DHAP were phosphofructokinase Pfk1/2 and fructose-1,6-bisphosphate aldolase Fba1, and therefore, the endogenous pathway has D-xylulose-1-phosphate as an intermediate rather than D-ribulose-1-phosphate. EG was formed from glycolaldehyde via endogenous alcohol dehydrogenase activity. Interestingly, a yeast strain with no D-xylulose kinase activity that overexpressed only phosphofructokinase PFK1/2 produced the highest amount of EG (about 2.8 g/L from 50 g/L D-xylose in bioreactor). This amount was further increased to over 4 g/L by fed-batch fermentation with D-glucose feed, which also increased the viability of the producer strain. The discovery of GA as a by-product of the EG-producing strains also indicates that this pathway could be modified for GA production in yeast. The authors speculate that for the endogenous pathway to work, the strain has to have sufficient D-xylose isomerase activity but no D-xylulose kinase activity (Uranukul et al. 2018).

### Glycolic acid and ethylene glycol production via the Dahms pathway

In the native Dahms pathway found from *Caulobacter crescentus*, D-xylose is first oxidised by D-xylose dehydrogenase to D-xylonolactone. D-xylonolactone is hydrolysed to D-xylonic acid either spontaneously or with the aid of a lactonase (Dahms 1974; Stephens et al. 2007; Nygård et al. 2014). D-Xylonic acid is then dehydrated by D-xylonate dehydratase to 2-keto-3-deoxyxylonic acid (2K3DXA) that can be further converted to pyruvate and glycolaldehyde by an aldolase. Glycolaldehyde can be either reduced to EG or oxidised to GA (Fig. 4). Unlike X1P, R1P and LX1P, this pathway produces pyruvate instead of DHAP, and although it does not use ATP for substrate phosphorylation, it is energetically less efficient because gluconeogenesis is needed for the growth.

Liu et al. (2013) deleted the native D-xylose isomerisation pathway and used the Dahms pathway including D-xylose dehydrogenase (Xdh) from *C. crescentus* and endogenous D-xylonate dehydratases and aldolases for EG production in *E. coli*. The resulting EG titer was 11.7 g/L (70% of the theoretical yield), but carbon was lost to GA and deletion of *aldA*-encoding aldehyde dehydrogenase converting glycolaldehyde to GA led to accumulation of D-xylonate. In a more recent study, the toxic effect of D-xylonate accumulation was alleviated by controlling *xdh* expression through a weak promoter and EG production from glycolaldehyde was improved by expressing a more efficient aldehyde reductase, *yjgB*. This resulted in EG titer of 1.52 g/L with a yield up to 98% of the theoretical yield (Cabulong et al. 2017). Wang et al. 2018 improved the redox balance of the pathway and employed FucO using NADH as a coenzyme to convert glycoaldehyde into EG. Additionally, to eliminate the production of GA and acetate, *aldA* and *arcA* were deleted. The resulting *E. coli* strain accumulated 72 g/L EG, with the yield of 0.40 g/g D-xylose in the fed-batch bioreactor cultivation (Wang et al. 2018). The strain differed from most of the other published strains with the Dahms pathway in that D-xylose isomerase-encoding gene from the competing native D-xylose catabolic pathway was not deleted. Chae et al. (2018) used the same strategy in construction of *E. coli* strain that produced 108.2 g/L of EG in a fed-batch fermentation on D-xylose minimal medium with the yield and productivity of 0.36 g/g and 2.25 g/L/h, respectively. Following the selection of the best *E. coli* strain and glycolaldehyde reductase (YqhD) for EG production, in silico genome-scale metabolic simulation was used to optimise the fluxes through the native D-xylose catabolic and the Dahms pathways. Interestingly, the highest EG productivity and titer were obtained by increasing the biomass formation. This was done by reduction of the *yqhD* expression and by increasing the flux through the native D-xylose catabolic pathway through downregulation of the Dahms pathway. Drawback of this strategy was that increased biomass production competed for the carbon source with EG production and thus decreased the EG yield (Chae et al. 2018).

In order to produce GA in *E. coli*, the Dahms pathway was combined with the expression of GS and two reverse glyoxylate pathway enzymes malate thiokinase that converts malate to malyl-CoA and malyl-CoA lyase that cleaves malyl-CoA to acetyl-CoA and glyoxylate (Cabulong et al. 2018) (Fig. 1b). The work included deletion of the glycolate oxidase-encoding gene to prevent GA consumption by *E. coli* and optimisation of the Dahms pathway by testing and selecting of the best combination from D-xylonate dehydratase and 2K3DXA aldolase enzymes. The best strain with *xdh*, *yagFE* and *aldA* overexpressed produced ~4 g/L GA. The strain engineering continued by activation of GS and reverse glyoxylate pathway by overexpression of *aceA*, *aceK*, *sucCD-2*, *ycdW* and *mcl1*. In combination with the Dahms pathway, overexpression of *aceA* and *aceK* was sufficient to slightly improve GA production up to 4.57 g/L GA with a yield of 0.46 g/g from D-xylose (Cabulong et al. 2018). The Dahms pathway in combination with the TCA cycle and GS pathways was also used for production of precursors for the production of poly(lactate-co-glycolate) (PLGA) biopolymer in *E. coli* (Choi et al. 2016, 2017).

The expression of genes encoding D-xylose dehydrogenase (XylB) and D-xylonate dehydratase (XylD) from *C. crescentus* and YagE or YjhH aldolase and aldehyde dehydrogenase (AldA) from *E. coli* in yeast *S. cerevisiae* enabled GA production from D-xylose up to 150 mg/L (Salusjärvi et al. 2017). In addition, 14 mg/L EG was produced due to reduction of glycolaldehyde by an endogenous enzyme activity of yeast. GA production was further increased up to 1 g/L by additional overexpression of lactate dehydrogenase encoding gene (Salusjärvi et al. 2017). Yeast, unlike *E. coli*, tolerates substantial amount of D-xylonate but its accumulation due to low activity of XylD in yeast limited EG and GA yield (Toivari et al. 2010; Andberg et al. 2016; Salusjärvi et al. 2017). Moreover, carbon was lost as 3-deoxypentonic acid (Salusjärvi et al. 2017).

### Serine pathway

Serine can be converted to glycolaldehyde either (i) via deamination of serine to hydroxypyruvate by aminotransaminase or amino acid dehydrogenase and decarboxylation of hydroxypyruvate to glycoaldehyde by α-ketoacid decarboxylase or (ii) via decarboxylation of serine to ethanolamine by serine decarboxylase and oxidation of ethanolamine to glycoaldehyde by monoamineoxidase (Chen et al. 2017) (Fig. 5). Glycoaldehyde can then be either reduced to EG by alcohol dehydrogenase or oxidised to GA by aldehyde dehydrogenase. The former pathway was used for EG production in *E. coli* with the additional metabolic modifications including overexpression of the biosynthetic pathway of L-serine and deletion of enzyme activities consuming the intermediates of the pathway. As a result, 4.1 g/L of EG at a cumulative yield of 0.14 g/g D-glucose was produced (Pereira et al. 2016b). Chen et al. 2017 tested the both routes in *C. glutamicum* strain with enhanced L-serine biosynthesis separately and in combination. The routes starting with serine deamination or decarboxylation resulted in EG titers 0.7 g/L and 1.7 g/L, respectively when cells were grown on minimal medium with D-glucose. Combination of both routes resulted in EG production of 3.5 g/L (0.09 g/g D-glucose) in batch bioreactor cultivation.

## Theoretical comparison of different routes for glycolic acid and ethylene glycol production

Microorganisms do not naturally produce GA or EG under typical growth conditions, and therefore, their biotechnological production requires metabolic engineering of a high-flux pathway from industrially relevant carbon sources. The so far demonstrated metabolic routes for their production utilise either D-glucose, D-xylose, D-arabinose, L-lyxose, L-arabinose, acetate or ethanol as carbon sources. GA production through the glyoxylate shunt is versatile in terms of carbon sources as any substrate metabolised to pyruvate can be used. In this pathway, one carbon is lost in the decarboxylation of pyruvate to acetyl-CoA and therefore, the theoretical yields for the biosynthesis of GA from D-glucose or D-xylose via GS are 2 mol/mol and 1.66 mol/mol, respectively or 1 mol/mol when ethanol is used as a carbon source (Koivistoinen et al. 2013) (Table 2). From D-glucose and D-xylose the theoretical GA yield of GS pathway is nevertheless higher than from the other D-xylose based pathways. The native metabolic flux to pyruvate is generally high but the challenge in the engineering of GS pathway is in optimisation and balancing the fluxes of the TCA cycle. In order to channel carbon to glyoxylate the conversion of isocitrate to alpha-ketoglutarate and glyoxylate to malate have to be blocked that reduces fitness of the cells. The problems may also arise from the fact that D-glucose represses the activity of the TCA cycle that makes it necessary to engineer also the D-glucose repression mechanisms of the cells. As an example, *REG1* involved in regulation of D-glucose repressible genes was deleted from *S. cerevisiae* in order to increase the activity of isocitrate lyase (Koivistoinen et al. 2013). In *E. coli*, deletions of repressors *iclR* or *arcA* have been used to attenuate repression of glyoxylate shunt or TCA cycle genes, respectively (Alkim et al. 2016). The additional obstacle is that expression of the NADP(H)-dependent glyoxylate reductase causes a redox imbalance that needs to be balanced elsewhere in the metabolism. This results in formation of by-products, e.g. lactate or acetate. Still another by-product, succinate is formed when isocitrate is cleaved to glyoxylate and succinate by isocitrate lyase. The reactions of the TCA cycle can convert succinate to oxaloacetate and further back to glyoxylate. However, in yeast, this may be hindered by the mitochondrial localisation of these enzymes while GS reactions take place in cytosol. Deng et al. 2018 reported recently GA production of 90% of the theoretical yield with *E. coli* strain with modifications addressing the most of the aforementioned obstacles of GS. However, the GA titer 65 g/L and rate 0.85 g/L/h need to be improved for viable biotechnological process.

**Table 2:** Theoretical yields of glycolic acid (GA) and ethylene glycol (EG) from different biosynthetic pathways used for GA and EG production

Pathway	Product	Carbon source	Theoretical yield (mol/mol)	Theoretical yield (g/g)
Glyoxylate shunt	GA	D-glucose	2	0.84
		D-xylose	1.66	0.84
		Ethanol	1	1.65
D-xylulose-1-phosphate	GA	D-xylose	1	0.51
	EG	D-xylose	1	0.41
D-xylulose-1-phosphate + glyoxylate shunt	GA	D-xylose	2	1.0
D-ribulose-1-phosphate	GA	D-xylose	1	0.51
	EG	D-xylose	1	0.41
D-ribulose-1-phosphate + glyoxylate shunt	GA	D-xylose	2	1.0
L-xylulose-1-phosphate	GA	L-arabinose	1	0.51
	EG	L-arabinose	1	0.41
L-xylulose-1-phosphate + glyoxylate shunt	GA	L-arabinose	2	1.0
Dahms	GA	D-xylose	1	0.51
	EG	D-xylose	1	0.41
Dahms + glyoxylate shunt	GA	D-xylose	2	1.0
Serine	GA	D-glucose	2	0.84
	EG	D-glucose	2	0.69

GA can be produced from different carbon sources also by the serine pathway, as the starting point of this pathway is the glycolytic intermediate 3-phosphoglycerate. The pathway produces glycolaldehyde and therefore also EG can be produced. The theoretical yields from D-glucose are similar to GS pathway, 2 mol/mol. Also in this pathway, one carbon is lost in decarboxylation reaction and therefore, every D-glucose molecule can only produce two molecules of 2-carbon EG or GA at maximum. The pathway does not utilise ATP and it is redox neutral for EG production. If GA is the intended product, NAD^+^ is produced in excess. The pathway has been used so far only for EG production, and the reported yields remained low, only 20% of the theoretical (Table 1) (Pereira et al. 2016b). Construction of a highly efficient strain based on the serine pathway may be challenging due to the lack of known enzymes that would efficiently catalyse the reactions of the pathway, especially enzymes from serine to glycolaldehyde are poorly characterised. Moreover, serine has an important role in several different metabolic pathways. Therefore, it is not possible to block all side reactions of the pathway making it difficult to divert a strong carbon flux towards a product.

X1P, LX1P and R1P pentose utilisation pathways all rely on aldolases to cleave 5-carbon sugar or sugar phosphate into 2-carbon glycolaldehyde and 3-carbon (DHAP) intermediates. The pathways are simple and energetically favourable; 1 ATP and 1 NAD^+^ are consumed by the pathway while 1 NADH and 2 ATP produced from DHAP in glycolysis. In *E. coli* with these pathways, yields close to the theoretical have been achieved for both GA and EG (Table 1) (Alkim et al. 2015; Pereira et al. 2016a; Cam et al. 2016). The drawback is that it is not possible to convert D-glucose, the most abundant sugar in biomass, to product via these pathways. These pathways are also less efficient in terms of theoretical yield than GS or the serine pathway because only glycolaldehyde can be readily utilised for GA or EG production (Table 2). Attempts to direct also the remaining 3-carbon molecule to GA via GS increased the GA yield but not the titer and reduced the production rate substantially (Table 1) (Pereira et al. 2016a). EG can be produced only from glycolaldehyde, while the means for transferring the 3-carbon intermediate into EG are lacking. Thus, the maximum yield of EG is only 1.0 mol/mol pentose (or 0.4 C-mol/C-mol) by these pathways, which is not economically feasible for industrial production of EG.

The Dahms pathway provides GA or EG 1 mol/mol D-xylose or GA 2 mol/mol D-xylose depending on whether pyruvate is converted to GA via GS or not (Table 2). The pathway is redox neutral for EG production but excess NADH is formed when GA is produced. In *E. coli*, GA and EG yield over 90% of theoretical that have been achieved by the Dahms pathway but titers remained relatively low (> 12 g/L) (Table 1) (Cabulong et al. 2017, 2018). In a recent study Chae et al. 2018 achieved substantially higher EG titer (108 g/L) and production rate (2.25 g/L/h) in fed-batch fermentation with *E. coli* when part of the carbon was allowed to go for growth. This titer and rate are already close to commercially feasible figures, considering that productivity of biotechnological EG should be higher than 100 g/L, 0.5 g/g and 3.0 g/L/h to be competitive with chemical synthesis (Zhang et al. 2017).

## Conclusions and future prospects

Due to the environmental issues, there is an increasing demand for production of platform chemicals like GA and EG from renewable resources by sustainable biotechnological processes. GA and EG are chemicals with a relatively low price, and therefore, the development of efficient production processes with a low cost is highly important for their commercialisation. Consequently, it is important to design potential metabolic routes that enable utilisation of preferably several carbon sources at the same time from various cheap and renewable feedstocks. These include for example industrial and agricultural waste streams like lignocellulosic sugars or starch from non-edible plants. However, recently pathways for EG production were designed even for gaseous substrates CO_2_, CO and H_2_ (Islam et al. 2017), although these routes have not yet been tested in practice. In practice, the pathway should also produce GA or EG at high theoretical yield. None of the existing pathways fulfills these criteria entirely and especially GA and EG production titers remain with most pathways too low for commercial production. The best reported EG production figures achieved with the Dahms pathway in *E. coli* are, however, already close to be competitive with its chemical synthesis. It would be highly interesting to test whether the same strain engineering strategy would also enable GA production via the Dahms pathway with the similar titer and rate. In general, metabolic engineering of *E. coli* for GA or EG production has been more successful than engineering yeast species. More efforts have not only been put in engineering of *E. coli* but also the enzymes of the GA and EG pathways have turned out be more difficult to express in yeast. Improved pathway enzyme activities, balanced enzyme expression and balance between the growth and the metabolic fluxes towards product seem to be of key importance for further improving GA and EG in metabolically engineered microbes. The use of genetic circuits that sense the metabolic needs of both the growth and the product formation would perhaps be an option for future research efforts.
